# Make African grasslands climate-change resilient

**DOI:** 10.1038/s43247-025-02109-3

**Published:** 2025-02-14

**Authors:** Tersur T. Akpensuen, Andrew D. Cartmill, Simón Pérez-Márquez, Helen Sheridan, Michael R. F. Lee, M. Jordana Rivero

**Affiliations:** 1 Net Zero and Resilient Farming, Rothamsted Research, North Wyke, Devon, EX20 2SB United Kingdom; 2 Department of Animal Production, Faculty of Agriculture, University of Jos, 93001 Jos, Nigeria; 3 School of Agriculture and Environment, Massey University, Palmerston North, 4442 New Zealand; 4 School of Agriculture and Food Science, University College, Dublin 4, D04 V1W8 Ireland; 5 School of Sustainable Food and Farming, Harper Adams University, Newport, Shropshire TF10 8NB UK

**Keywords:** Agriculture, Climate-change mitigation

## Abstract

Climate change has negatively impacted grassland productivity in Africa. Climate-smart technologies such as forage grass, legume, and herb mixtures could enhance grassland productivity and resilience, offering a sustainable solution for African pasture-based livestock systems.

Grasslands (intensive and extensive) are Africa’s dominant land use type, accounting for 44.8% of the total land area^1^, which provides feed for livestock and wild animals. Approximately 70% of people in rural Africa depend on livestock for their livelihoods^2^. As human populations increase, grasslands are increasingly being transformed into arable land and other uses. The remaining grasslands often experience overgrazing due to livestock production, resulting in significant land degradation. This is exacerbated by climate change, with shifting weather patterns and increased frequency of extreme weather events (e.g. drought and flooding), the spread of invasive species, and bush encroachment. Consequently, there is a significant reduction in forage quality and quantity, increased livestock disease vulnerability, and mortality rates, threatening regional food security. Given the importance of livestock production to smallholder farmers’ livelihoods in Africa^3^, we believe in adopting sustainable practices that could enhance the productivity of intensively and extensively managed African grasslands for economic, social, and environmental benefits. The Global Farm Platform initiative, a community of collaborative practitioners investigating sustainable ruminant livestock systems around the globe (www.globalfarmplatform.org), highlighted management strategies for sustainable livestock systems^4^. Here, we argue that various forage species mixtures could enhance the sustainability of agricultural grasslands in Africa.

## Sustainable intensification of grasslands

Sustainable intensification is an approach that aims to produce more food output while efficiently utilizing natural resources and reducing negative impacts on the environment. The approach has been adopted as a policy goal for several national and international organisations focused on sustainable development goals^5^. The policy goal of sustainable intensification also extends to research on grassland and pasture systems, and it was reflected in the theme of the International Grassland Congress (New Delhi, India, November 2015): *Sustainable Use of Grassland Resources for Forage Production, Biodiversity, and Environmental Protection*. This goal is essential for managing tropical grasslands, which is vital to reverse land degradation that could promote regional food security and livelihoods.

One effective strategy for sustainable intensification in grassland systems involves using multispecies swards. Multispecies swards are mixtures of three or more forage species selected for their agronomic benefits and complementary interactions^6^. It is a mixture of grasses, legumes, and herbs vital for improving farming systems’ multifunctionality, resilience, and sustainability. Multispecies swards have been applied to intensive grassland management with encouraging results in terms of ecosystem service delivery in temperate regions. There are also ongoing multi-site intercontinental studies across temperate regions like the Legacy Net project investigating the benefits of multispecies swards to improve quality and quantity of forage yields, resource use efficiency, ecosystem service provision, and the legacy effect of the multispecies swards in subsequent crop rotation systems^7^. However, there are research gaps related to the use of multispecies swards in Africa with most studies focusing on monocultures or binary mixtures. Deliberate reseeding of African grasslands with multispecies swards could boost forage quality and quantity while addressing soil degradation to prevent the environmental mistakes associated with industrialised agriculture. The use of monocultures that require excessive use of fertilizers and herbicides has led to environmental degradation. Shifting to multispecies swards could mitigate these impacts, fostering resilient and eco-friendly grassland systems.

## Summary benefits of multispecies swards in grasslands

Multispecies swards can reduce the impact of extreme weather events like droughts and floods and improve tolerance to stresses linked to climate change^7^. For instance, deep-rooted species access deeper water during droughts, while shallow-rooted species benefit from rainfall^8^, supporting productivity. Multispecies swards promote soil health, improve water retention, reduce soil erosion, and carbon and nitrogen footprints^9^. Using multispecies swards in intensively managed grassland systems could boost biodiversity, which is essential for ecosystem stability^10^. Biodiversity ensures ecosystem stability by allowing other species to fill ecological niches if one is affected by pests or diseases, a vital function amid climate change, which can alter pest and disease patterns. Legumes in forage species mixtures enhance pollinator and earthworm populations^11^ and enrich the soil by fixing nitrogen reducing synthetic fertilizer use^5^. Integrating different agronomically beneficial plant species can increase forage biomass and quality^12^ and improve animal performance compared to monocultures^13^.

## Global Farm Platform’s Considerations for a Climate-Change Resilient African Grasslands

**Fig. 1:**
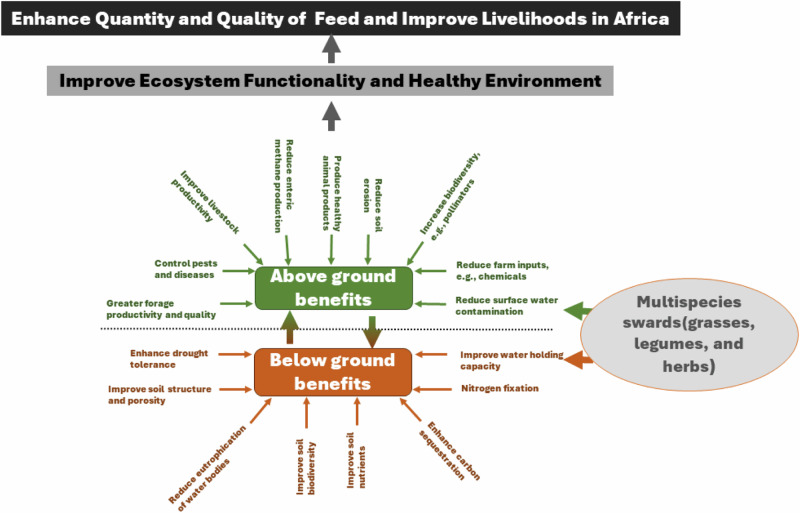
Schematic benefits of multispecies swards to transform grasslands systems, resilient and sustainable livelihoods in Africa.

Despite the numerous benefits of multispecies swards, there are challenges to their adoption and implementation in Africa. One of the primary challenges is the lack of knowledge and technical expertise for the selection and evaluation of multispecies sward forage mixtures of tropical-origin species generally. While there is a substantial body of evidence regarding the benefits of multispecies swards to help address many societal concerns regarding the sustainability of livestock production systems in temperate regions, there is a paucity of information regarding their benefits in Africa. Careful selection of species (grasses, legumes, and herbs) for their agronomic benefits can result in an interconnected system both above and below ground, providing mutual benefits, with the ultimate advantage being a sustainable and productive agroecosystem in Africa (Fig. 1). Climate, topography, soil types, land use, production system, and management, among other considerations, vary in different regions influencing the productivity of forage species^14^. As members of the Global Farm Platform, we believe sustainable intensive grassland systems are achievable in Africa through multispecies swards. This could lead to enhanced environmental sustainability of livestock production systems with livelihood improvement at the local level in various African agro-climatic conditions.

Given the current environmental concerns, adequate funding for research in Africa and tropical countries is important. The preferred option is to form research consortia like those in Europe and other continents in temperate regions such as the LegacyNet (https://legacynet.scss.tcd.ie/) and globally like that of the Global Farm Platform. We highlight research needs in grassland-based livestock agriculture in Africa and tropical regions in general, focusing on multispecies sward forage mixtures:

- **Assess soil health in multispecies swards versus high-input monocultures in Africa**. Multispecies swards include nitrogen-fixing legumes and deep-rooted plants, which naturally improve soil fertility and structure, and reduce the need for farm inputs like fertilizers. This is particularly important in Africa, where most farmers are smallholders with little investment.
- **Evaluate the carbon sequestration and climate resilience of tropical forage mixtures versus monocultures in Africa**. Multispecies swards can be more resilient to extreme climate events, sustaining productivity and carbon sequestration. Resilient forage mixtures stabilise carbon storage and aid in understanding how land management can optimize sequestration while promoting sustainable grasslands in Africa.
- **Evaluate the impact of multispecies swards on soil greenhouse gas emissions and enteric methane emissions in African tropical grasslands**. Multispecies swards may reduce grassland and livestock-origin greenhouse gas emissions. It is important to understand the role of tropical multi-forage species mixtures in mitigation strategies for greenhouse gas emissions from intensively managed grasslands in Africa.
- **Study the impact of multispecies swards on the performance of livestock and biomass productivity in Africa**. Research has demonstrated that multispecies can enhance higher biomass yield and stability with lower nitrogen input. This can support livestock productivity making them an attractive option for farmers.
- **Evaluate the impact of multispecies swards on livestock health in Africa**. Forages with natural anthelmintic properties reduce reliance on synthetic dewormers, helping to mitigate issues like drug resistance in parasites, a growing global concern. Researching forage mixtures suited for Africa could provide an understanding of selecting forage with both nutritional value and health-promoting properties while having reduced parasite burdens.
- **Assess the nutritional quality of animal products from multispecies swards to enhance human nutrition in Africa**. The limited availability of quality animal products remains a major concern, especially for low-income households across many African regions. Incorporating multispecies swards into dairy production systems could improve milk production and quality compared to grass monocultures. Understanding the role of tropical African forage mixtures is essential for quantifying the nutritional quality of animal products in Africa.
- **Assess the adaptability of multispecies swards in different agro-climatic zones of Africa**. The adaptability of forage species varies in different climatic conditions. Informed forage selection from research studies is vital to give farmers region-specific guidance on which combinations work best in their areas, enhancing their ability to make informed pasture establishment and grazing decisions for the best result outcomes.

Multispecies swards represent a promising climate-smart strategy for resilient grassland systems which could be vital to achieving more sustainable and resilient African grassland and ruminant production systems. By leveraging the complementary and/or facilitatory interactions between different plant species, these systems can potentially withstand the challenges posed by climate change while providing nutritious food for humans, supporting local livelihoods, and enhancing biodiversity and associated ecosystem service provision. However successful implementation depends on closing knowledge gaps in areas such as species selection, adaptability, fertilization practices, defoliation intensity and frequency, and understanding their economic value in different agroclimatic regions. With the right support and investment, multispecies grasslands could be crucial for sustainable land management in Africa and underpin local and global climate resilience.
